# “A Piece of Sanity in the Midst of Insane Times”: *Girls on the Run* Programming to Promote Physical Activity and Psychosocial Well-Being During the COVID-19 Pandemic

**DOI:** 10.3389/fpubh.2021.729291

**Published:** 2021-10-11

**Authors:** Maureen R. Weiss, Lindsay E. Kipp, Allison Riley

**Affiliations:** ^1^School of Kinesiology, University of Minnesota, Minneapolis, MN, United States; ^2^Department of Health and Human Performance, Texas State University, San Marcos, TX, United States; ^3^Girls on the Run International, Charlotte, NC, United States

**Keywords:** positive youth development, out-of-school-time, youth sport, social-emotional learning, holistic health

## Abstract

Afterschool programs have the potential to promote social, emotional, and physical health outcomes among youth participants. The positive youth development (PYD) framework argues that acquiring desirable attitudes and behaviors occurs when skill-building opportunities are explicitly provided within a safe and supportive climate guided by caring, competent, and compassionate instructors. *Girls on the Run* (GOTR) is a PYD program that uses running, motor skills, and other physical activities as a platform for promoting positive psychosocial outcomes and life skills learning among elementary- and middle school-aged girls. The onset of the COVID-19 pandemic challenged GOTR to modify lessons, coach training, and program delivery (in-person, virtual, or hybrid) to accommodate public health guidelines. The purpose of this study was to assess caregivers' and coaches' perceptions of program effectiveness in light of these changes. Following the Fall 2020 season, caregivers (*n* = 1,617) and coaches (*n* = 991) from 1,077 teams and 39 councils completed an online survey about program experiences. Both stakeholder groups positively rated program impact regardless of delivery mode, although in-person mode was rated higher for satisfaction with the end-of-season event. Thematic analysis of open-ended responses revealed that caregivers and coaches identified increased physical activity opportunities and life skills learning as well as improved social, psychological, and emotional development as a result of participating. Both stakeholders noted GOTR provided a sense of normalcy during this time of great need. Findings using mixed methods provide evidence of program effectiveness and recommendations for youth programming during challenging times.

## Introduction

*Girls on the Run* (www.girlsontherun.org) is a physical activity-based positive youth development (PA-PYD) program using running, motor skills, and physical activities as a platform for promoting psychosocial development and life skills among girls 8–11 (grades 3–5) and 12–14 years (grades 6–8). The program typically serves 200,000 girls annually and engages 50,000 coaches (98% female). The intentional curriculum and systematic coach training are aligned with best practices for PYD programs, including opportunities for skill building, a safe physical and psychological space, appropriate structure, supportive relationships, and feelings of belonging (1, 2).

The program adopts Lerner's Five *Cs* framework to guide lessons comprising the life skills curriculum (3). Lessons are designed to help girls develop social, emotional, and physical *competence*, feel *confident* in who they are, create positive *connections* with peers and adults, develop strength of *character*, and respond to others and self with *care* and compassion. A sixth C reflects *contribution* to the greater good through implementing a community service project. The curriculum for 3rd−5th grade girls focuses on three themes: identity (self-care and self-awareness), connectedness (selecting and maintaining healthy relationships), and empowerment (celebrating and sharing one's strengths), while the 6th−8th grade curriculum focuses on lessons related to self, team, and community. Sessions also prepare girls to complete the culminating 5 K, by setting goals, regulating progress, and participating in a practice 5 K to build confidence. To attain program goals, girls and coaches meet twice a week for 75–90 min over a 10-week season. Coaches are trained to deliver the curriculum with fidelity, along with an emphasis on building relationships, creating a positive, inclusive environment, and emphasizing a mastery climate. More information about GOTR is available in other sources [e.g., (4–6)].

Evidence of program effectiveness shows GOTR's impact on physical activity, life skills, and holistic health [e.g., (7–10)]. Pre- to post-season improvements emerged for activity levels, self-esteem, physical competence, and peer support; these were retained 3 months after season's end. Life skills transfer for managing emotions, helping others, resolving conflicts, and decision-making favorably compared to girls in organized sport and school physical education. Qualitative data from girls, caregivers, coaches, and school personnel indicated that girls improved in social and emotional behaviors and physical activity motivation as a result of participating in GOTR.

The onset of the COVID-19 pandemic challenged GOTR to modify program delivery to accommodate public health guidelines, while continuing to promote PYD in a time of great need. The curriculum was shortened from 20 to 16 lessons and delivery options included in-person, virtual, or hybrid. Health and safety policies were adopted utilizing CDC guidance for all modes. When in person, coaches delivered pre-COVID lessons with modifications for physical distancing (e.g., “air” high fives, shadow tag) and the use of new journals outlined in a curriculum addendum. Girls also used the journals in the virtual space. If a team was 100% virtual or needed to transition to the virtual space (i.e., hybrid), coaches used the newly developed Virtual Lesson Curriculum, which contained lessons that mirrored the in-person learning objectives. Virtual lessons were shortened to 45–60 min plus a 20–30-min independent workout (modified from 75–90 min in-person lessons), and a variety of physical activities were provided to be inclusive and allow for space constraints (modified from in-person workouts that primarily included running and other locomotor skills). The workout typically completed in person was transitioned to a “separate but together workout” that girls started together at the end of the lesson and continued on their own. Coach training, typically delivered in-person (4.5 h) and online (1 h), was moved to completely online. Three training modules were added: impact of COVID-19, coaching virtually, and coaching for social inclusion. The end-of-season 5 K, typically a large community celebration, became a “K Your Way” event.

Consistent with GOTR's commitment to ongoing evaluation for improving curricula and program delivery, the organization developed an online survey to seek caregivers' and coaches' perceptions of program effectiveness in light of COVID-19 protocol changes. Based on utilization-focused evaluation, Patton (11) accentuates bridging empirical research and practical implications of evaluation findings. He suggests that evaluations provide answers to three questions: (1) *What* information emerges about attitudes, skills, and behaviors? (2) *So What* do findings imply about program effectiveness? (3) *Now What* recommendations can be made for making program improvements? The purpose of this study was to answer these questions based on stakeholders' perceptions of COVID-19 safety protocol changes to program delivery.

## Method

Following the Fall 2020 season, all GOTR councils (~200) were invited to evaluate the curricular and delivery modifications due to COVID-19 restrictions. Thirty-nine councils elected to participate, consisting of 1,077 teams in all regions of the U.S. GOTR national headquarters distributed an online survey to all caregivers and coaches within the 39 councils.

### Caregivers

A total of 1,617 caregivers completed the survey. They reported their girls' program delivery mode as virtual (23.3%), in-person (54.3%), and hybrid (22.4%). For girls' race/ethnicity, 73% were white/Caucasian, 7% Black/African American, 7% multiracial, 6% Latina, 3% Asian, 0.4% American Indian/Alaskan Native, 0.3% Native Hawaiian/Pacific Islander, and 1% Other (~2% did not say). Most girls (91%) were in grades 3–5 and 9% were in grades 6–8.

### Coaches

A total of 991 coaches completed the survey. They delivered the program as virtual (32.1%), in-person (47.3%), and hybrid (20.6%). The majority were white/Caucasian (84%), with 4% Black/African American, 3% Asian, 3% Latina, 3% multiracial, 0.3% American Indian/Alaskan Native, 0.1% Native Hawaiian/Pacific Islander, and 1% Other (~2% did not say). Most coaches were between 25–44 years old (61%); 39% were first-timers with GOTR, while 61% coached for ≥2 years.

### Survey Questions

For caregivers, 5 statements probed level of agreement on the GOTR experience (Table 1), ranging from strongly disagree (1) to strongly agree (5). Another question sought degree of satisfaction with the end-of-season event, ranging from completely dissatisfied (1) to completely satisfied (5). They also rated “how true” a statement was (“my girl feels lonely”, “my girl is confident”, “my girl is physically active”) before and after participation in GOTR, ranging from not true at all (1) to really true (5). Coaches responded to 8 statements probing level of agreement about their GOTR experience and 3 questions about how satisfied they were with the COVID-19 safety protocols, end-of-season event, and communication from GOTR (Table 2).

**Table 1:** ANOVAs for caregiver items by delivery mode: *F-* and *p-*values, means, standard deviations, and effect sizes.

**Survey question:**	***F-*** **value (*p-*value)**	***M*** (***SD***)			**Cohen's** ***d***	
		**Virtual**	**In-person**	**Hybrid**	**Virtual vs. in-person**	**Virtual vs. Hybrid**
GOTR has been a valuable experience for my girl.	^*^19.73 (*p* < 0.001)	4.48 (0.77)	4.73 (0.61)	4.68 (0.63)	0.33	0.26
GOTR helped my girl gain skills that are helping her handle the stress associated with the pandemic.	4.80 (*p* = 0.008)	4.24 (0.83)	4.38 (0.73)	4.37 (0.77)	—	—
GOTR has led to at least one conversation with my girl about an important topic.	0.78 (*p* = 0.46)	4.23 (0.89)	4.27 (0.83)	4.31 (0.81)	—	—
Because of participating in GOTR, my child is more confident.	^*^9.54 (*p* < 0.001)	4.07 (0.87)	4.28 (0.75)	4.25 (0.77)	0.24	0.21
The COVID-19 precautions in place gave me confidence that GOTR was striving to create a safe experience for my girl.	1.15 (*p* = 0.28)	—	4.56 (0.85)	4.51 (0.81)	—	—
How satisfied were you with the end-of-season event?	^*^34.09 (*p* < 0.001)	4.02 (1.11)	4.54 (0.97)	4.43 (1.02)	0.47	0.37
Before vs. after GOTR — My girl feels lonely.	4.65 (*p* = 0.01)	−0.49 (0.99)	−0.69 (1.05)	−0.63 (1.06)	—	—
Before vs. after GOTR — I would describe my girl as confident.	0.22 (*p* = 0.80)	0.51 (0.84)	0.54 (0.77)	0.54 (0.74)	—	—
Before vs. after GOTR — My girl is physically active.	1.56 (*p* = 0.21)	0.66 (0.93)	0.76 (0.94)	0.70 (1.01)	—	—

*All responses were on a 1–5 scale. A p-value of <0.006 was deemed a significant effect, based on a Bonferroni adjustment (0.05/9 items).*

* *Denotes significant effect. Cohen's d was calculated for statistically significant differences. No statistically significant differences emerged for In-Person vs. Hybrid delivery modes. Change score indicates “after minus before”, thus caregivers in all three delivery modes reported their girls decreased in loneliness and increased in confidence and physical activity*.

**Table 2:** ANOVAs for coach items by delivery mode: *F-* and *p-*values, means, standard deviations, and effect sizes.

**Survey question:**	***F-*** **value (*p-*value)**	***M*** (***SD***)			**Cohens** ***d***		
		**Virtual**	**In-person**	**Hybrid**	**Virtual vs. in-person**	**Virtual vs. hybrid**	**In-person vs. hybrid**
Coaching Girls on the Run has been a valuable experience for me.	^*^5.95 (*p* = 0.003)	4.77 (0.45)	4.77 (0.47)	4.63 (0.64)	–	−0.32	−0.22
I formed positive relationships with the girls on my team.	^*^8.88 (*p* < 0.001)	4.55 (0.60)	4.73 (0.51)	4.66 (0.55)	0.29	0.18	–
Girls on my team developed positive relationships with their teammates.	^*^24.77 (*p* < 0.001)	4.31 (0.75)	4.65 (0.55)	4.55 (0.58)	0.44	0.32	–
As a Girls on the Run coach, I felt like I was making a difference in girls' lives.	2.40 (*p* = 0.09)	4.48 (0.61)	4.57 (0.62)	4.50 (0.60)	–	–	–
Because of participating in Girls on the Run, girls on my team are more confident.	4.76 (*p* = 0.009)	4.39 (0.63)	4.53 (0.63)	4.44 (0.60)	–	–	–
I received sufficient training to effectively implement the program.	1.34 (*p* = 0.26)	4.42 (0.66)	4.48 (0.67)	4.41 (0.71)	–	–	–
Coach support was available to me throughout the season.	1.21 (*p* = 0.30)	4.65 (0.57)	4.58 (0.66)	4.59 (0.61)	–	–	–
I had the support I needed to coach during COVID times.	0.16 (*p* = 0.86)	4.54 (0.63)	4.57 (0.64)	4.55 (0.65)	–	–	–
How satisfied were you with the COVID-19 related safety protocols implemented this season?	0.01 (*p* = 0.92)	–	4.50 (0.99)	4.51 (0.99)	–	–	–
How satisfied were you with the end-of-season event?	^*^34.67 (*p* < 0.001)	3.85 (1.09)	4.48 (0.95)	4.33 (1.00)	0.58	0.44	–
How satisfied were you with the communication from Girls on the Run?	0.26 (*p* = 0.77)	4.62 (0.84)	4.66 (0.81)	4.65 (0.79)	–	–	–

*All responses were on a 1–5 scale. A p-value of <0.005 was deemed a significant effect, based on a Bonferroni adjustment (0.05/11 items).*

* *Denotes significant effect. Cohen's d was calculated for statistically significant differences*.

One open ended question for each stakeholder was selected for qualitative analysis based on relevance to the COVID-19 protocol changes. For caregivers, “Share one way that your girl has been positively impacted by her *Girls on the Run* experience, such as something she learned, a favorite moment or activity, or a lasting takeaway.” For coaches, “This is a challenging time for many girls. What do you think was the greatest need met through the program during this time?”

### Data Analysis

Quantitative responses were analyzed using analysis of variance to determine delivery mode differences. We applied a Bonferroni adjustment to avoid Type 1 errors (12). For caregivers, statistical significance was determined using *p* < 0.006 (0.05/9 items) and for coaches using *p* < 0.005 (0.05/11 items). Student-Newman-Keuls *post-hoc* tests were used in the event of statistically significant differences. Effect size (ES) was calculated for statistically significant differences using Cohen's *d* (13): *d* ≥ 0.20 = small, ≥0.50 = medium, ≥0.80 = large.

An inductive content analysis was adopted for the qualitative responses (14). One researcher initially coded data units (words, phrases, sentences) and lower-order themes for half the sample of caregivers (*n* = 780) and coaches (*n* = 458) due to early saturation of responses. A second researcher randomly selected 100 responses for each stakeholder and independently coded data units and lower-order themes. The researchers then met to discuss convergence and divergence of findings and came to consensus. Together they derived a set of higher-order themes that emerged from the lower-order themes and data units (14).

## Results

### Caregivers: Quantitative Responses

Responses (Table 1) fell between agree and strongly agree regardless of delivery mode (virtual, in-person, hybrid). Caregivers favorably viewed the COVID-19 safety precautions in the in-person and hybrid modes. Analysis revealed no differences for: (a) gained skills for handling stress, (b) participating led to at least one conversation between caregiver and girl about an important topic and (c) COVID-19 precautions gave confidence that GOTR was striving to create a safe experience for girls. Two items showed a difference favoring in-person vs. virtual and hybrid vs. virtual—valuable experience and my girl is more confident. ES's were small. No statistical differences emerged for in-person vs. hybrid delivery modes.

Caregivers reported the location of their end-of-season event as in-person at site (72.3%), virtual (22.0%) or other (5.7%). Satisfaction ratings with the event fell between somewhat and completely satisfied for all delivery modes, but caregivers of girls who participated in virtual sessions scored significantly lower than girls who experienced in-person or hybrid lessons. ES's were small.

For the before and after questions —“my girl felt/feels lonely”, “I would describe my girl as confident”, and “my girl was/is physically active”—scores improved for all three delivery modes. Caregivers reported girls as less lonely, more confident, and more physically active. No differences in change scores emerged by delivery mode.

### Coaches: Quantitative Responses

Responses (Table 2) fell between agree and strongly agree regardless of program delivery. Coaches reported positive experiences across the board, including high ratings for “coach support was available to me throughout the season” and “I had the support I needed during COVID times.” Coaches favorably viewed COVID-19 safety protocols for in-person and hybrid modes. No differences emerged for: (a) received sufficient training to effectively implement the program (b) as a GOTR coach, I felt like I was making a difference in girls' lives, and (c) because of participating in Girls on the Run, girls on my team are more confident.

In-person delivery received more favorable scores than virtual delivery for: I formed positive relationships with girls on my team and girls developed positive relationships with teammates; these items were also higher for hybrid vs. virtual. All ES's were small. When asked if coaching GOTR has been a valuable experience, coaches in the hybrid mode scored significantly lower than virtual and in-person modes, but ES's were small.

Coaches reported location of the end-of-season event as in-person at site (62.5%), virtual (28.7%) or other (8.9%). Satisfaction ratings with the event were significantly higher for coaches who delivered the program in-person or hybrid than for virtual mode. ES's were small (virtual vs. hybrid) and medium (virtual vs. in-person). No differences emerged between delivery modes for satisfaction with safety protocols and support and communication from GOTR.

### Caregivers: Qualitative Themes

Coding responses to, “Share one way that your girl has been positively impacted by her GOTR experiences …” resulted in 9 higher-order themes derived from 15 lower-order themes and hundreds of data units. Table 3 depicts the thematic analysis.

**Table 3:** Caregiver responses: lower-order themes and data units within each higher-order theme.

**Lower-order theme:**	**Data units:**
**Higher-order theme: improved physical activity motivation and behavior**	
Physical activity opportunities	Learning to be more physically active Becoming more active Discovered she loves running as an activity Outdoor exercise Looked forward to running Running has gotten better Loves running with her friends Found she is good at running More active in the outside than before Understands how important daily physical activity is to her well-being Energetic from the workout Training for, committing to, and completing a 5 k Wants to run track now Excited about exercise Understands the value of being active She has a new love for running Enjoys the movement activities during each session The exercise helped give her balance with the virtual school day She's focusing on fitness Being able to run longer distances Learning to be active by running Helped her gain physical strength as she recovered from an injury Lots of fun exercise Love to run Now enjoys exercising Learned that she really enjoys running and that she can do it My daughter values physical activity as a way to release stress Getting more physical activity She has better endurance
**Higherorder theme: social development**	
Building friendships	Making new friends (numerous entries) Formed long-lasting friendships Connected with friends Seeing friends virtually or in person Time spent with friends Brought her closer to girls she otherwise would not have been able to get to know Made lifelong friends that she calls her sisters
Opportunity for social interactions	Interacting with girls More outgoing Came out of shell Conversations with teammates Helped her open up more Positive interactions with her peers
Feeling a part of the group/team	Team spirit Sense of community Feeling of belonging Group activities Being part of a team and lifting each other up Made her feel like a welcome part of a group Built a strong camaraderie with the other girls Loved how everyone cheered and uplifted each other A fun sense of belonging Team building Bonding with the other girls
**Higher-order theme: psychological growth**	
Achieving Goals	Striving toward a goal such as 5 k Setting goal for 5 k
	Meeting/exceeding goal for 5 k Pushed self to achieve running goals Got better at accomplishing goals Felt a strong sense of achievement after running the 5 k She was able to complete it (5k) without stopping and felt successful in her efforts Able to prove to herself that she could do more than she initially thought possible Learned that she can push herself to accomplish big goals
Gaining confidence	Willingness to try new things More confident about running Gained confidence Now confident enough to volunteer to speak in group Zoom meetings She has the confidence that she can tackle any challenges Confidence in running the 5 k
	Confidence demonstrated by her growing comfort in engaging in new activities Confidence with being a leader She doesn't doubt herself so much anymore on the first try Now has the mindset that girls can be strong and do anything! More confident with exercising She knows she can do hard things
**Higher-order theme: emotional growth**	
Being able to express feelings	Talk about feelings More open More outspoken Learned how to better communicate her feelings More willing to share and speak publicly Learned to make her voice heard Voices her opinion more Talking about her feelings when she gets frustrated Using positive words to speak with those who upset her, or she disagrees with She is more articulate about her emotions More open to talk to me Expressing her feelings Talk about emotions more freely She has emotionally matured since doing GOTR
Adopting a positive outlook	Positive thinking Being optimistic Using positive self-talk Positive attitude Motivated Using positive language about herself Learning about positivity and being uplifting A lot of positive encouragement Positive self-statements/positive thinking skills Learned how to think more positively
Feeling proud of accomplishments	Sense of pride for finishing 5 k Proud of overcome a hard physical task (5 k) Proud to have run as far as she did Proud of herself to finish her 5 k her way Proud she completed the 5 k without quitting Pride in running longer distances
**Higher-order theme: life skills development**	
Learning life lessons	Star Power (learned how to activate her Star Power) Strategies to cope with stress Choosing good friends Learned to enjoy the success of others rather than only celebrating her own achievements
	Used some of the lessons to work through problems with her little sister Learning about making healthy choices Understanding of empathy Tries to brainstorm ways to positively problem solve when a problem arises Coping skills for stressful situations Looking out for your friends Learned to be more of a leader Learned more about her feelings and some things to do to make her feel better when she's anxious Gave her a chance to develop a positive body image She has a better understanding of true friendships and how to cope with life's challenges Emphasis on heathy lifestyle both physically and mentally Learned dedication, positive thinking and improved self-esteem Toolbox for navigating emotions, peer relationships, and social dynamics Helped my daughter socially, emotionally, and physically She's aware of her health choices and mental break options in keeping herself happy Gained quite a bit of empathy Improving social skills Learned how to communicate more clearly She learned she is able to push herself beyond what she was able to do before. Learned strategies for helping her stay calm
Helping others	Learned about giving back Community service project Sense of civic engagement Donating to charities Encouraging teammates Providing words of encouragement to the other girls Thinking about her communities Helping her community Community project helped her feel empathy for others Be supportive for each other Ran extra laps with her friends in order to support them
**Higher-order theme: positive coaching influence**	
Positive coaching influence	Trusted coaches Coaches were positive role models Loved her coach Encouragement she gets from the coaches Interacting with adults outside the family Having coaches who were engaged and interested in listening Enjoyed the positive feedback and encouragement from her leaders Felt safe in speaking her opinion and that's cause the leaders fostered that environment
**Higher-order theme: closer family connections**	
Closer family connections	Stronger connections with parents and siblings Running together
	Discuss lessons with mother Going on a weekly jog as a family Running with her mom at the 5 k More positive and patient with her family We have been taking daily walks as a family to remind us all to be more active This has been something we could do together She had some hilarious conversations that gave the entire family a laugh—something we all very much need right now! She came home one day from GOTR and we had a conversation about how girls apologize too much, and I LOVED this conversation!
**Higher-order theme: having fun**	
Having fun	Enjoy the program Enjoy running Enjoy the girls Being silly Having fun in the program
**Higher-order theme: sense of normalcy during the pandemic**	
Sense of normalcy during the pandemic	Gave her something to look forward to each week Getting outside Being with girls aside from virtual school Allowed for safe and positive interaction with schoolmates Spending time with a small group of girls in a comfortable and honest setting Made her feel like she was living some normal moments. Grateful to have a covid-safe option when so many activities are canceled She appreciated the time to do something NORMAL in this not-normal year Having a social and active outlet during pandemic GOTR experience helped her feel much more connected to the school community at a time of stress and loneliness

Improved Physical Activity Motivation and Behavior was evident in data units revealing opportunities for being physically active with teammates and looking forward to running and training for the 5 K. As one parent said, “My daughter has grown to love running and wants to run with me now. She sees herself as a runner and this is a positive identity that she will carry with her into the future.” Another parent shared, “My daughter did not like to do any physical activity prior to this program. After GOTR, she would come home in such a positive mood and feeling better about herself. Now she is pushing me to incorporate exercise into our lives and strives to be a healthy person.”

Social Development was characterized by opportunities to develop and strengthen friendships, socially interact with peers, and experience group belongingness—various ways that allowed girls to share common bonds with others their age. One parent offered, “This was a wonderful way for Sadie^1^ to get reconnected with kids her age and have some meaningful experiences in the midst of this COVID chaos. Thank you for providing a safe and supportive way for her to be with other kids and continue working on her running skills.” This parent gave a more vivid example, “My daughter became motivated and dedicated to completing her own physical fitness routine because of the positive energy and camaraderie generated by her GOTR coaches and team. She felt she was part of something important, a little bit more connected to others and appreciated as a person.”

Psychological Growth was defined by themes of achieving goals and gaining confidence. Caregivers attested to girls setting goals and developing strategies to successfully complete them (e.g., 5 K). Others highlighted how confidence was enhanced through activities and lessons in GOTR. One parent shared, “My daughter was really struggling and feeling alone during COVID. She lost her motivation and happy spirit … After just one practice she was happy again. She was eager to set goals and achieve them. As she achieved goals her confidence grew. This program was even more important this year!”

Emotional Growth entailed themes of being able to express feelings, adopting a positive outlook, and feeling proud of accomplishments. Responses alluded to becoming more open with communicating, engaging in positive talk, and feeling a sense of pride in completing the 5 K. A parent shared, “She was really struggling with COVID and not being able to be with her friends … when she started GOTR back up she was excited, she looked forward to each session, it brought her back to life and her outgoing self again.” Another offered, “GOTR gave her peers to interact with … she was able to share her thoughts on the lesson without hesitation. GOTR has always been a positive, uplifting experience where she feels accepted.”

Life Skills Development was prominent, characterized by learning life lessons and helping others. Caregivers confirmed that girls acquired specific “tools” (Take a Breather) and learned important skills (e.g., choosing good friends) that transferred to many areas of girls' lives. A parent shared, “The lessons have been so helpful for Pria. We have gone back to lessons again and again and generalized for everyday life. Positive self-talk and Take a Breather helped us get through things like the flu shot and a medical procedure that was pretty scary. As a child with anxiety … having those tools in her toolkit helped us more than I can express in words.” Helping Others was a theme reinforcing lessons that emphasize standing up for others (being a “Stand-Byer”) and giving back to community and society. Developing empathy and a sense of civic engagement is accentuated in the curriculum through completion of a community service project.

Two higher-order themes revolved around meaningful adult relationships, Positive Coaching Influence and Closer Family Connections. Caregivers were uniformly praiseworthy of their girls' coaches as positive role models, sources of encouragement, and fostering a safe and inclusive climate. In addition, several caregivers commented on how girls' involvement in GOTR fostered opportunities for being physically active as a family and having conversations related to lesson themes. Having Fun was a theme that reflected the sheer enjoyment of running, being with peers, and experiencing the overall program.

A Sense of Normalcy during the Pandemic emerged as directly relevant to the COVID-19 pandemic. Caregivers praised the value of the program in light of virtual school and other social activities being canceled. One parent summarized concisely, “She appreciated the time to do something NORMAL in this not-normal year.” Another shared, “My daughter was new to her school and with it being virtual, she hadn't met anyone. Her GOTR experience helped her feel much more connected to the school community at a time of stress and loneliness.”

### Coaches: Qualitative Themes

Coding responses to, “… What do you think was the greatest need met through the program during this time” resulted in 7 higher-order themes derived from 9 lower-order themes and hundreds of data units. Table 4 depicts the thematic analysis.

**Table 4:** Coach responses: lower-order themes and data units within each higher-order theme.

**Lower-order theme:**	**Data units:**
**Higher order theme: increased physical activity**	
Opportunities to be physically active	Physical activity Not only were they active during the lesson, but it continued at home too Able to get outside and exercise More active Being able to do the 5 k Movement Participate in an outdoor activity with friends Exercise and moving their bodies Time to move Ability to be outside exercising with other girls Opportunity to get outside and move Got to be active which they don't do during online learning Opportunity to be active with others Getting exercise Giving them a lot of moving time Keeping girls active Opportunity to enjoy their love for running following the safety guidelines Able to participate in a sport/activity Exercise outdoors Encouraged them to get up and MOVE! Getting outdoors for physical activity A chance to be active Opportunity to have an outlet for activity
**Higher-order theme: social development**	
Socializing with peers	Meaningful social interaction Connecting with teammates Social and peer interaction Need for social and emotional interaction A place to be sociable and silly Opportunity to connect with peers in a fun and safe way Time to socialize with peers Chance to engage with others in a positive manner Building relationships and being able to spend time with peers Created socialization opportunities Fellowship with others Interactions with peers See their friends Make new friends Forming relationships Forming better friendships and getting along well Allowing girls to socialize Connecting through positive experiences
Feeling part of a group/team	Still able to have a team experience A sense of community Feeling like part of a group Awareness that they were not alone Togetherness Belonging to a team Enjoyed the companionship that they received during practice Opportunity to participate in a “group” activity Teamwork and unity Need of togetherness Belonging A sense of connectedness with girls of the same age group
**Higher-order theme: psychological growth**	
Coping with stress	Mental health Managing negative feelings
	Cope with the world at the moment Allowed them to de-stress and focus on things outside the stress of everyday life Learn skills to cope with this stressful, constantly changing time Teaching girls coping skills Discussed coping skills for the stress of virtual school and sadness Strategies to cope with stress Spending time furthering their mental health needs
Building confidence	Reinforcing their confidence Sense of accomplishment after completing the 5 k Building confidence in what their bodies can do Opportunity to put some stress aside and become a stronger and more confident girl A chance to focus on their self-development Watched their confidence grow in expressing themselves
**Higher-order theme: life skills development**	
Learning life lessons	Positive mindset Opportunity to develop social skills (sharing, cooperation, overcoming shyness, productive disagreement and conflict resolution) Having lessons on emotions bridges gaps with how they are feeling and how they can express it The lessons about feelings and how to manage and communicate them were very helpful Participate in an outdoor activity with friends where they were learning important life skills Staying Positive Opportunity to learn about self-love and respect for others Developed skills to see their strengths, push themselves to reach their goal, and how to support each other Positive self-talk Learned tools which are very helpful to use in their everyday lives, especially during a time like now How to manage emotions Focusing on the positive Learning that people that bring you up are better to be around than people that bring you down Learning social skills “Star power” to build self confidence Lessons that incorporated feelings and how to handle them Learn great life skills during a difficult time Practicing coping methods for negative feelings and self-talk Able to build a toolbox of skills through the lessons Develop strong habits/practices in exercise, mental health, confidence, and relationships Team building skills Learning important life skills during a hard year Star Power
**Higher-order theme: positive social environment**	
Having a safe and supportive environment	Safety for the girls and coaches Safe, multipurpose, extracurricular activity A safe and healthy environment for the girls to discuss issues important to them Positive, meaningful, and real connection that kept safety the top priority Supporting each other Having a safe place to go where they could release some energy in a positive way Forming relationships with other girls in a positive and supportive environment
	Creating an environment for girls to support each other Having a safe and open space for them to share their thoughts and ideas Have meaningful discussions around topics being dealt with in daily lives A chance to talk about Covid in a safe setting Girls had a platform to share in their joys, excitement, triumphs, and hardships Time for girls to talk about their feelings Opportunities to chat about what was on their mind. Time for girls to talk about their feelings in a safe space A place where girls can be silly, a little less formal, and express themselves Opportunity for the girls to talk about their feelings The girls had exactly what they needed to participate safely Providing a safe space for girls to vent and enjoy time with one another A safe space to express emotions Gave them a sense of security Able to express their emotions Being able to talk with other people their age Surrounded by tons of positivity from girls their own age Allowed them to express themselves A safe place to express emotions and share experiences
**Higher-order theme: having fun**	
Having fun	Provided a sense of fun Enjoyed having a more laid back, social activity A fun way for the girls to connect Participate in fun games to take their mind off of things A place for girls to have fun Fun interaction and engagement
**Higher-order theme: sense of normalcy during the pandemic**	
Sense of normalcy during the pandemic	A piece of sanity in the midst of insane times Feeling a sense of normalcy and belonging Surrounded by their peers gave them some normalcy Gave the girls a sense of calm in all this chaos Focusing on growth and positivity during such a limiting and negative time was wonderful for them Great way to get girls moving during this pandemic where you have to spend a majority of time indoors Giving them something to look forward to during this time For many, this was their first time getting together with people outside of their family since before COVID Ability to have a routine Provided girls with stability during uncertain times Chance to be with other girls and stay active during this period of isolation Giving the girls something to look forward to twice a week Consistency—allow girls to continue participating in a meaningful program they enjoy and look forward to. Giving the girl something positive to do during this hard time The girls were able to be “normal” during our time together. Getting back to something somewhat normal! A feeling of normalcy–when so much has been canceled including almost all clubs, GOTR was there. A distraction from the pandemic

Some themes were similar to those of caregivers, revolving around opportunities to socialize with peers, be physically active, build confidence, learn life lessons, be surrounded by a positive social environment, and feel a sense of normalcy. One coach nicely summarized how GOTR met girls' needs during this challenging time: “Giving girls the opportunity to get out of the house and develop strong habits/practices in exercise, mental health, confidence, and relationships.”

Numerous responses exemplified the theme, Increased Physical Activity, including opportunities to be physically active by getting outside, participating in sports and activities with friends, and as one coach implored, “Encouraged them to get up and MOVE!” Another shared, “Many parents told us that before GOTR, their girls were not getting any physical activity. Not only were they active during the lesson, but it continued at home too.” As a PA-PYD program, GOTR is unique in emphasizing physical activity as a healthy behavior through opportunities to enjoyably experience running, motor skills, and movement activities that are continued outside of the structured program.

Social Development entailed themes of socializing with peers and feeling part of a group. Coaches highlighted meaningful interactions and relationships as a crucial need that was achieved through activities promoting teamwork, unity, connectedness, and companionship. One coach shared, “The program provided the girls an opportunity to meet and interact with other girls and school staff (coaches). This gave the girls a much-needed opportunity to socialize with peers/adults during the difficult time caused by COVID (school closures, social distancing).” Another added, “I think social interaction was the greatest need. Girls had a platform to share in their joys, excitement, triumphs and hardships. My girls made valuable connections and even exchanged numbers to keep in touch with one another.”

Psychological Growth included lower-order themes of coping with stress and building confidence. Coaches elaborated on how GOTR met these needs, “It gave the girls a sense of calm in all this chaos”, “Having lessons on emotions bridges gaps with how they are feeling and how they can express it”, and “They just needed someone besides people in their immediate households to talk with and to explore the world around them with different people so that they weren't stuck stressing about school. They got the opportunity to put some stress aside to have fun and to become a stronger and more confident girl.”

Life Skills Development was characterized by learning life lessons, such as adopting a positive mindset, managing emotions, developing social skills, and helping others. One coach explained, “… the opportunity to develop social skills (including sharing, cooperation, overcoming shyness, productive disagreement and conflict resolution), given that many aren't in school.” Another said, “Many of the girls developed skills to help them see their strengths, push themselves to reach their goal, and how to support each other.” One elaborated: “The girls were able to build a toolbox of skills through the lessons that helped them in this current odd environment … The girls learned that other girls felt the same way about life/the world. There was safety in knowing they were not alone.”

Positive Social Environment was exemplified by having a safe and supportive space for engaging in activities and interacting with peers. A coach stated, “GOTR gave the girls something to look forward to and people to interact with. I think most of them were lonely and hadn't seen any peers in a while. GOTR gave them a space to talk about their lives and how they've been feeling.” Other coaches stated, “This made connection possible outside of the household. Positive, meaningful, and real connection that kept safety the top priority”, and “The program was able to help with providing a safe place for girls to connect while they are experiencing isolation due to the pandemic … the content was encouraging and helped to keep them positive and growing during a challenging time.” The higher-order theme, Having Fun, also reflected a positive and supportive climate—enjoying activities with peers and fun games to take their mind off things (i.e., COVID).

Sense of Normalcy during the Pandemic depicted a theme specific to meeting girls' needs during COVID-19. One coach succinctly characterized GOTR's role, “A piece of sanity in the midst of insane times.” Another exclaimed, “Getting back to something somewhat normal! For the time we were together, besides masks, distancing and sanitizer, it felt good to do something other than be on the computer at home all day.” Another stated, “Normalcy. So many of our girls had things interrupted over the last 8 months and to bring the program back at our school was so helpful and nice to see them involved in an after-school activity”.

## Discussion

Utilization-focused evaluation research is a means for bridging evidence-based findings with practical implications (11). It begins with the premise, “… evaluations should be judged by their utility and actual use … how real people in the real world apply evaluation findings” (p. 4). Patton states that evaluation research should answer three questions: *What* … changes in attitudes, skills, and behaviors occur in participants? *So What* … do the findings imply about the degree to which the program is considered a success? *Now What* … recommendations flow from the findings? We systematically address study findings relative to these questions based on caregivers' and coaches' perceptions of safety protocol changes to curricula and delivery mode.

*What* changes in girls' attitudes, skills, and behaviors occurred by participating in GOTR? Quantitative responses by caregivers and coaches were favorable (e.g., my girl learned skills to handle stress associated with the pandemic), regardless of the curriculum delivered in-person, virtually, or hybrid. Caregivers reported girls as less lonely, more confident, and more physically active at season's end—for all modes. Qualitative themes provided detail for how girls were impacted by GOTR (caregivers) and what greatest need was met by participating during this challenging time (coaches). Based on these data, GOTR provided a means of sustaining physical activity, strengthening friendships, achieving goals, and building confidence. Girls acquired ability to express feelings, maintain optimism, and take pride in completing the 5 K. Evidence of season-long improvement in physical and psychosocial outcomes align with the mission, vision, and core values of the program (5) as well as longitudinal findings during “normal” program delivery (9, 10).

Qualitative data also revealed that learning life skills and feeling a sense of normalcy were central to countering the challenges girls faced, by acquiring skills to cope with stress, think positively, and develop relationships (6). Stakeholder groups credited GOTR with teaching life lessons that informed intentional decision-making (positive self-talk, choosing friends), managing negative emotions, and adopting healthy behaviors to navigate many areas of girls' lives during the pandemic. Ability to generalize lessons learned in GOTR to domains outside the program—school, family, peers—is a signature of PYD programs (2, 6).

*So What* … do findings imply about the degree to which GOTR was effective? Mixed methods revealed improvement in girls' physical activity and psychosocial attitudes and behaviors that infer program success using varied delivery modes and safety precautions. These promising outcomes occurred within a safe and supportive social environment that included encouraging coaches and meaningful peer interactions. “Social distancing” was not equivalent to reduced opportunities for socializing with peers, developing social skills, and experiencing a sense of community—innovative ways of delivering the program were successful in promoting PYD. Peer acceptance and close friendships are critical needs for youth in childhood and adolescence (15, 16), which was achieved through GOTR's quality programming and coach training. Building positive relationships is a key element of coach training, attained through activities that highlight cooperation, inclusion, support, and a mastery climate (5, 17). As a PA-PYD program challenged with modifying curricular lessons to accommodate safety precautions, GOTR was successful in providing an environment that made a positive impact on girls' physical activity and psychosocial and emotional growth and development.

*Now What* … recommendations can be made for program improvements? While favorable ratings emerged for all delivery modes, the virtual experience was rated lower for a few items (albeit small effect sizes). This included, “GOTR has been a valuable experience for my girl”, “I formed positive relationships with girls on my team”, and “girls on my team developed positive relationships with teammates.” Satisfaction with a virtual end-of-season event was rated lowest. GOTR used the findings to recommend areas for improving the Spring 2021 season. First, councils were encouraged to conduct the end-of-season 5 K in person and at site-based locations if possible. Second, to optimize developing positive relationships, virtual session length was extended from 45 to 60 min and ideas were added to the virtual curriculum for coaches to make more informal connections. Third, a team workout option was added to the virtual curriculum (girls and coaches complete it together), which increased time for connection and motivation for physical activity.

Despite strengths of this study (e.g., mixed methods, multiple stakeholders), we note some limitations. First, 39 councils agreed to participate and, although large samples of caregivers and coaches from over 1,000 teams completed the survey, it is unknown how those in other councils viewed the program experience. Second, the sample was not as diverse as the makeup of the broader organization. Survey responses represented 25% girls of color, whereas the network composition for Fall 2020 was 37%. GOTR remains committed to gathering insights from individuals of diverse sociocultural backgrounds and is pursuing strategies to ensure representation. Third, to maximize return rate the survey length was kept reasonable, which did not allow for more quantitative items. The open-ended questions provided rich data to complement ratings and lent perspective on areas of holistic health and well-being attained through participating.

In conclusion, GOTR resourcefully and effectively applied COVID-19 safety precautions with in-person, virtual, and hybrid modes. All modes were received favorably and open-ended narrative revealed the breadth and depth of program impact. Evaluation findings provide GOTR and other youth programs with critical information for improving curricula, coach training, and program delivery while maintaining high safety protocols during a challenging health crisis.

## Data Availability Statement

The datasets presented in this article are not readily available because the dataset is proprietary to Girls on the Run International. Requests about the data should be directed to Maureen Weiss, mrweiss@umn.edu.

## Ethics Statement

The project was reviewed by Research Integrity and Compliance (RIC), Texas State University, San Marcos, TX, USA. Because the study exclusively involved the examination of anonymous, secondary data, the research is not regulated by RIC, and written informed consent for participation was not required.

## Author Contributions

MW and AR conceived the study. AR collected the data. MW and LK drafted the earlier versions of the manuscript and conducted the qualitative data analysis. LK conducted the quantitative data analysis. All authors contributed to revising the manuscript and approved the final submitted version.

## Conflict of Interest

The authors declare that the research was conducted in the absence of any commercial or financial relationships that could be construed as a potential conflict of interest.

## Publisher's Note

All claims expressed in this article are solely those of the authors and do not necessarily represent those of their affiliated organizations, or those of the publisher, the editors and the reviewers. Any product that may be evaluated in this article, or claim that may be made by its manufacturer, is not guaranteed or endorsed by the publisher.

## Footnotes

^1^All names are pseudonyms.
